# Continuous quality improvement (CQI) Institutionalization to reach 95:95:95 HIV targets: a multicountry experience from the Global South

**DOI:** 10.1186/s12913-021-06731-7

**Published:** 2021-07-20

**Authors:** Peter Memiah, Josephine Tlale, Mope Shimabale, Sarah Nzyoka, Patience Komba, Jackson Sebeza, Adesina Tina, Violet Makokha

**Affiliations:** 1 Division of Epidemiology and Prevention, Institute of Human Virology, University of Maryland School of Medicine, 725 West Lombard Street, Room N345, MD 21201 Baltimore, USA; 2 Institute of Human Virology, Center for International Health, Education, and Biosecurity (CIHEB), University of Maryland School of Medicine in Botswana, Gaborone, Botswana; 3 Institute of Human Virology, Center for International Health, Education, and Biosecurity (CIHEB), University of Maryland School of Medicine in Zambia, Lusaka, Zambia; 4 Institute of Human Virology, Center for International Health, Education, and Biosecurity (CIHEB), University of Maryland School of Medicine in Kenya, Baltimore, Kenya; 5 Institute of Human Virology, Center for International Health, Education, and Biosecurity (CIHEB), University of Maryland School of Medicine in Tanzania, Baltimore, Tanzania; 6 Institute of Human Virology, Center for International Health, Education, and Biosecurity (CIHEB), University of Maryland School of Medicine in Rwanda, Kigali, Rwanda; 7 Institute of Human Virology, Center for International Health, Education, and Biosecurity (CIHEB), University of Maryland School of Medicine in Nigeria, Abuja, Nigeria

**Keywords:** Quality Improvement, HIV, Health Systems, Sustainability

## Abstract

**Background:**

Scaling up continuous quality improvement (CQI) processes could be key in achieving the 95:95:95 cascade and global HIV targets. This paper describes the experiences and outcomes related to implementing CQI processes to help reach these targets, with particular focus on clinical and programmatic settings in 6 countries from the global south.

**Methods:**

The HIV program at the University of Maryland, Baltimore (UMB) implemented an adapted CQI model in Kenya, Tanzania, Botswana, Zambia, Nigeria and Rwanda that included the following steps: (1) analysing the problem to identify goals and objectives for improvement; (2) developing individual changes or ‘change packages’, (3) developing a monitoring system to measure improvements; and (4) implementing and measuring changes through continuous ‘plan-do-study-act’ (PDSA) cycles. We describe country-level experiences related to implementing this adaptive design, a collaborative learning and scale-up/sustainability model that addresses the 95:95:95 global HIV targets via a CQI learning network, and mechanisms for fostering communication and the sharing of ideas and results; we describe trends both before and after model implementation.

**Results:**

Our selected country-level experiences based on implementing our CQI approach resulted in an increased partner testing acceptance rate from 21.7 to 48.2 % in Rwanda, which resulted in an increase in the HIV testing yield from 2.1 to 6.3 %. In Botswana, the overall linkage to treatment improved from 63 to 94 %, while in Kenya, the viral load testing uptake among paediatric and adolescent patients improved from 65 to 96 %, and the viral load suppression improved from 53 to 88 %.

**Conclusions:**

Adopting CQI processes is a useful approach for accelerating progress towards the attainment of the global 95:95:95 HIV targets. This paper also highlights the value of institutionalizing CQI processes and building the capacity of Ministry of Health (MoH) personnel in sub-Saharan Africa for the effective quality improvement of HIV programs and subsequent sustainability efforts.

## Background

In 2014, the world embarked on an ambitious goal to end the HIV epidemic by 2030 by adopting the 95:95:95 targets, which are also collectively known as the HIV fast-track approach [1]. These targets, developed by the Joint United Nations Program on HIV/AIDS (UNAIDS), seek to have 95 % of people infected with HIV diagnosed, 95 % of those diagnosed started on antiretroviral treatment (ART) and 95 % of those on treatment virally suppressed [2]. This fast-track approach is expected to avert nearly 28 million HIV infections and 21 million AIDS-related deaths by 2030, with a 15-fold return on HIV-related investments [2]. Key challenges to this approach, coupled with the differing nature of the epidemic on the global scale, include poor identification of undiagnosed HIV patients [3] and poor linkage and retention following diagnosis [4]. There are country variations in reaching these targets, with countries that are close to the target now focusing on improving their retention and viral suppression levels, whereas for other countries, significant investments are needed to mainly reach the priority populations [5].

Various approaches have been proposed to address these challenges, including quality improvement and quality assurance methodologies [6, 7]. Continuous quality improvement (CQI) is a method for improving health outcomes based on the use of routine program data to inform necessary changes [8]. CQI collaboratives adopted from the Institute of Healthcare Improvement’s Breakthrough Series are becoming an increasingly popular method for organizing sustained improvement efforts in low- and middle-income countries (LMICs) and as a strategy for improving the quality of services and patient care [9–11]. Through these collaboratives, CQI teams from multiple health facilities across a region or country are brought together to focus on a common health problem. One of the cornerstones of a CQI approach is the plan-do-study-act (PDSA) technique [12]. This technique involves identifying a problem, implementing minor alterations to processes and measuring the resulting changes to gauge the effectiveness of the new approach [13]. Its main objective is to quickly learn if a particular method produces the desired results and then make necessary changes to continuously improve the processes and outcomes [14].

Given the importance of an effective public health workforce, a central component of a CQI collaborative is enhancing the capacity of national and subnational health managers to act as CQI coaches who support the on-the-job training of healthcare workers and offer targeted mentorship [15]. The CQI coaches support the facility staffs to obtain and analyse their own facility-level data. The hands-on approach ensures skills transfer through site-based mentorship [16]. A key component of the CQI collaborative approach is regional or zonal performance review meetings comprising health workers from facilities participating in the initiative. During these meetings, data quality and progress is reviewed. The teams discuss strengths, weakness, and course correction planned. [17].

Various studies have shown improved HIV service provision after implementing a CQI approach [10]. A recent systematic review conducted by Hargreaves et al. [18] in LMICs reported an increase in the uptake of ART, adherence to treatment and viral load testing. However, there are significant disparities in the approaches, settings and reporting styles used, thereby making it challenging to identify best practices and to understand what specific aspects of these interventions lead to significant and sustainable clinical improvements in an LMIC context [18]. Although encouraging, these studies have not relied on national or subnational health managers and/or routine programmatic data to examine key indicators of the quality of care provided.

The success of meeting the 95:95:95 HIV targets in such settings using a CQI approach needs to be characterized based on the related effect on HIV diagnosis, the linkage to care and the subsequent initiation of ART, as well as viral load suppression. For these reasons, locally relevant evidence is needed to understand the effectiveness of CQI approaches in meeting these UNAIDS targets. The aim of this manuscript, therefore, is to describe CQI interventions that are implemented at the facility level using participatory, data-driven approaches, on-site monitoring and supervisory support. This study provides a description of the implemented CQI processes, as well as a pre-trend versus post-trend display of the effects of applying these approaches on improving HIV outcomes across the 95:95:95 cascade in six sub-Saharan countries.

## Methods

We applied the PDSA cycle in six sub-Saharan African countries, namely, Kenya, Tanzania, Botswana, Zambia, Nigeria and Rwanda. In these countries, UMB-led CQI teams applied the CQI process of analysing a situation to identify problems in the process; developing change ideas that would potentially address those problems; setting up or adopting a change package; testing small, measurable changes while monitoring and documenting every process; and reviewing data regularly to determine progress towards the improvement aim. In collaboration with Ministry of Health (MoH) staff members, the teams then decided whether to adopt, adapt, or discard a given change based on whether it resulted in desired improvements.

**Fig. 1:**
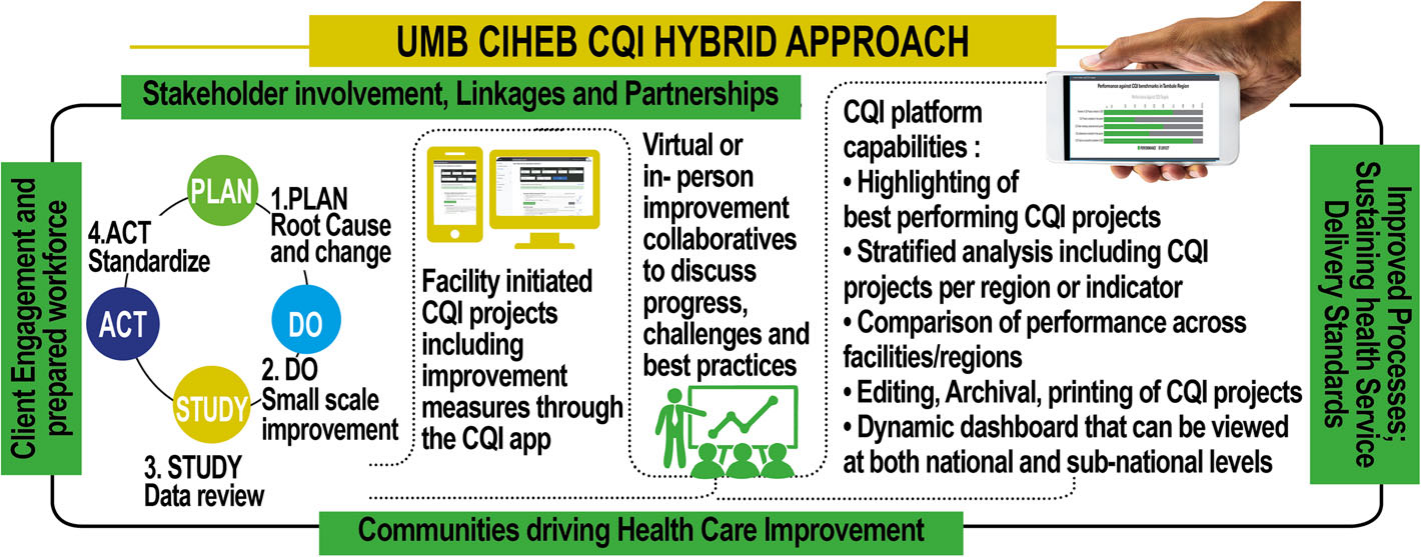
CQI tracking and implementation using the CQI digital platform

The type of support provided to MoH staff varied between countries; however, in general, the support consisted of a stepwise approach where the following occurred:

A 1-day sensitization course for top-level managers and leaders engaged in health service provision at the national and subnational levels led to the following:
(a) A five-day CQI didactic training course for trainers of trainers (TOTs), known as ‘CQI coaches’, at the subnational level, followed by the TOTs cascading their training to (b) a 5-day training course for CQI implementers (healthcare providers) at the facility level. The 1-day top-level managers’ sensitization course aimed at obtaining stakeholder buy-ins, resource mobilization and ownership. The 5-day training sessions involved didactic approaches, simulated exercises on the use of CQI tools and self-learning from UMB training materials that are now published on our website (www.ciheb.org/CQI).
On-site support from trained CQI coaches helped facility CQI teams apply the skills they learned from their training within their setting. The tracking of CQI projects was done through dynamic methods. Most recently, in Tanzania, Zambia Rwanda, Botswana and Kenya, we utilized an electronic CQI reporting system known as the CQI platform, which was developed by UMB (manuscript under publication). This platform has been integrated into tracking continuous quality improvement (CQI) projects for HIV/TB service delivery and provides users (CQI teams) with the real-time tracking of CQI projects, whereby CQI teams are shown to better self-manage their own QI with anecdotal evidence, which indicates that the platform increases work efficiency in regard to reporting CQI. Healthcare leaders at the subnational and national levels can use the CQI digital platform for the real-time tracking and monitoring of CQI projects and therefore provide immediate feedback to facility teams in regard to implementing CQI tracking, monitoring of CQI projects and providing feedback to facilities. The platform uses CQI PDSA qualitative and quantitative tools that have been subjected to Standards for Quality Improvement Reporting Excellence (SQUIRE) guidelines for the efficient electronic tracking of CQI initiatives with an inbuilt dynamic dashboard, as described in Fig. 1.

4. Finally, setting up face-to-face virtual learning collaboratives using platforms such as ECHO® or Zoom has also led to the development of CQI communities of practice (for Tanzania, Nigeria and Botswana).

This structured approach involved the CQI trainees working on multidisciplinary teams, picking specific improvement aims and using simple analytical tools to identify both causes for underperforming patient outcomes and iterative methods for testing and adapting solutions to improve patient care. The frequency of on-site support varied from country to country depending on the funding priorities, as specified by the donor. However, all high-volume facilities with over 1,000 patients undergoing HIV treatment received at least one quarterly visit from the coaches. Most of the follow-ups were conducted using the current administrative structures within the MoH subnational and regional teams.

In Kenya specifically, the process involved a county CQI focal person appointed by the County Director for Health who acted as a link between the county health management and the sub counties/facilities and led the county CQI technical working group (TWG). The county CQI focal person appointed subcounty focal persons who provided direct mentorship and the orientation of facility staff regarding CQI activities and conducted capacity building for implementers at the facility level. These individuals acted as a link between the subcounty and the county. At the facility level, the CQI coaches were facilitators of CQI knowledge and activities, and they oversaw the activities of the multidisciplinary CQI teams with support from the facility CQI team leaders. Facilities identified their areas of improvement based on their routine performance data. A system was established through the county TWG, whereby facilities came together on a semi-annual basis to share their best practices and lessons learned from the improvements that had been made. The UMB team also facilitated peer-to-peer learning sessions that allowed teams from different facilities to share their progress and learn from each other. Learning sessions were 2–3 days in length and were held either quarterly (in Tanzania and Zambia) or biannually (in Nigeria).

The six countries highlighted in this manuscript contribute the highest burden of HIV in the sub-Saharan region. Each country received funding from the United States President’s Emergency Plan for AIDS Relief (PEPFAR) to support HIV service delivery. Because different countries used different measures and carried out their work over different time periods, the data from each county were analysed separately. In the following, we present country case studies that describe the before and after changes in trends for different HIV outcomes along the 95:95:95 cascade, while fully describing the implementation process for each CQI project.

## Results

To illustrate the effect of implementing CQI processes, we present the key results found along the three UNAIDS cascade areas:

### Improved awareness of HIV status

To improve the first 95, we implemented CQI approaches that aimed to facilitate the identification of HIV-positive clients through partner notification services (PNS), otherwise known as the ‘testing of index partners’.

#### Case study 1

##### Problem

Index partner testing in 12 health facilities in Rwanda was below the target threshold of 80 % across the index testing cascade, i.e., the number of eligible people living with HIV (PLHIV) offered index partner testing services; the proportion of index cases who accepted index testing services; and the number of index partners contacted and tested for HIV.

##### Root cause analysis

The main problem identified included the poor training of health providers in regard to HIV testing, counselling and elicitation procedures.

##### Change idea(s) implemented

Between January and July 2019, facility CQI teams developed a change package and adjusted the work plans and indicator targets to meet the 80 % threshold. This package included the virtual training, mentorship and support of health providers. The teams also implemented intensive site-level monitoring combined with improved CQI capacity for facility-based health providers.

##### Outcome

**Fig. 2:**
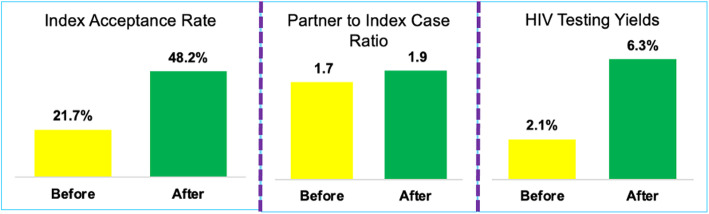
Index partner testing; Ratios and HIV testing yields in Rwanda

The index partner testing acceptance rate increased from 21.7 to 48.2 %, while the partner-to-index case testing ratio increased from 1.7 to 1.9. Using this approach, the HIV testing yield (defined as a percentage of the number of people who are HIV positive versus the number tested versus) increased from 2.1 to 6.3 % between January and July 2019 (Fig. 2).

#### Case study 2

##### Problem

Improving PNS in Kenya by supporting facilities in having > 90 % of patients whose partner(s) have been tested for HIV or know their HIV status.

##### Root cause analysis

The main problems identified included laborious manual reporting systems and the cross-training of facility staff on PNS processes with no follow-up of contacts.

##### Change idea(s) implemented

Between October 2018 and June 2019, we strengthened the documentation and monitoring of contacts and introduced counsellor performance monitoring with clear action points and ‘flexi’ hours to allow facility-based HIV testing counsellors to conduct community testing during off-peak times. We also introduced targeted community testing for clients who did not honour routine appointments and suggestion boxes within facilities for the anonymous identification of PNS contacts.

##### Outcome

**Fig. 3:**
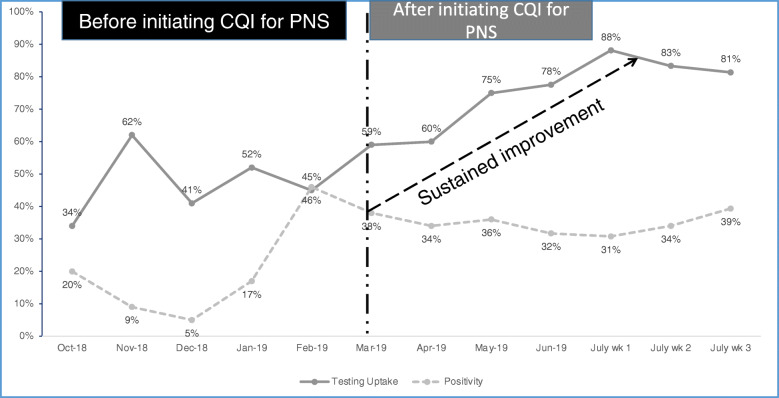
Improving partner notification services in Kenya

Index partner testing improved from a low of 45 % to a high of 80 % after 6 months, with observed upward trends over time (Fig. 3). These best practices were scaled up to all the sites, and the overall program performance was sustained above 80 %.

### Improved linkage and treatment in HIV care

To improve the second 95, we implemented CQI approaches that aimed to facilitate linkage and treatment in HIV care.

#### Case study 3

##### Problem

The linkage rates in 41 facilities in Botswana were below the target threshold of 95 %.

##### Root cause analysis

The main problems identified included human resource constraints that hindered ART initiation for HIV-positive clients identified after working hours; clients who tested HIV positive and then chose to initiate treatment at other facilities without any process to ensure follow-up; poor patient navigation regarding treatment initiation for those who tested positive; and the lack of a protocol to support HIV-positive clients who were not willing to initiate ART immediately.

##### Change idea(s) implemented

The introduction of a policy that allowed clients to be transferred to a preferred facility following ART initiation (i.e., Start and Transfer); the availability of 14-day starter packs of ART at multiple service delivery points in the health facility; the introduction of on-call clinicians both after-hours and weekends to initiate newly identified clients into treatment; the cross-training of facility staff with regard to testing and linking; the fast-tracking of peer counselling following HIV diagnosis; and collaboration with testing partners to build a cascade from testing to treatment, as well as the identification of champions to strengthen same-day ART initiation.

##### Findings

**Fig. 4:**
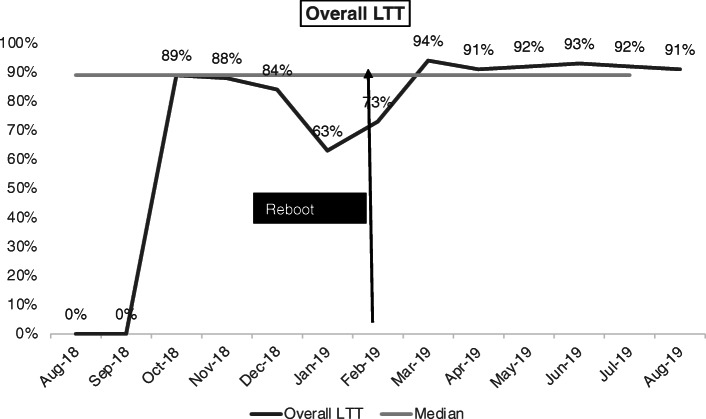
Improving overall linkage to treatment (LTT) in Botswana

**Fig. 5:**
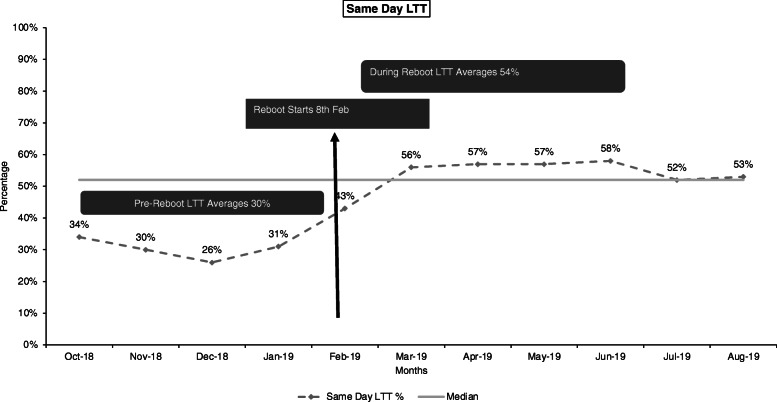
Improving same-day linkage to treatment in Botswana

Between August 2018 and August 2019, the overall linkage to treatment (LTT) improved from a low of 63–94 % and was sustained above 90 % over time (Fig. 4). Additionally, between October 2018 and August 2019, same-day LTT improved from a low of 26 % to a high of 54 % and remained above 50 % over time (Fig. 5).

### Improved viral suppression

To improve the third 95, we implemented CQI approaches that aimed to improve viral suppression.

#### Case study 4

##### Problem

Achieving viral suppression among HIV-infected paediatric and adolescent patients in Kenya has remained a challenge compared to adult suppression.

##### Root cause analysis

The main problems identified included the lack of a defined patient flow for clients who were due for viral load sample taking, the lack of a focal person to follow-up with viral load results from the laboratory, the lack of caregiver involvement/lack of steady caregivers and the lack of prompt verification for viral load results.

##### Change idea(s) implemented

**Fig. 6:**
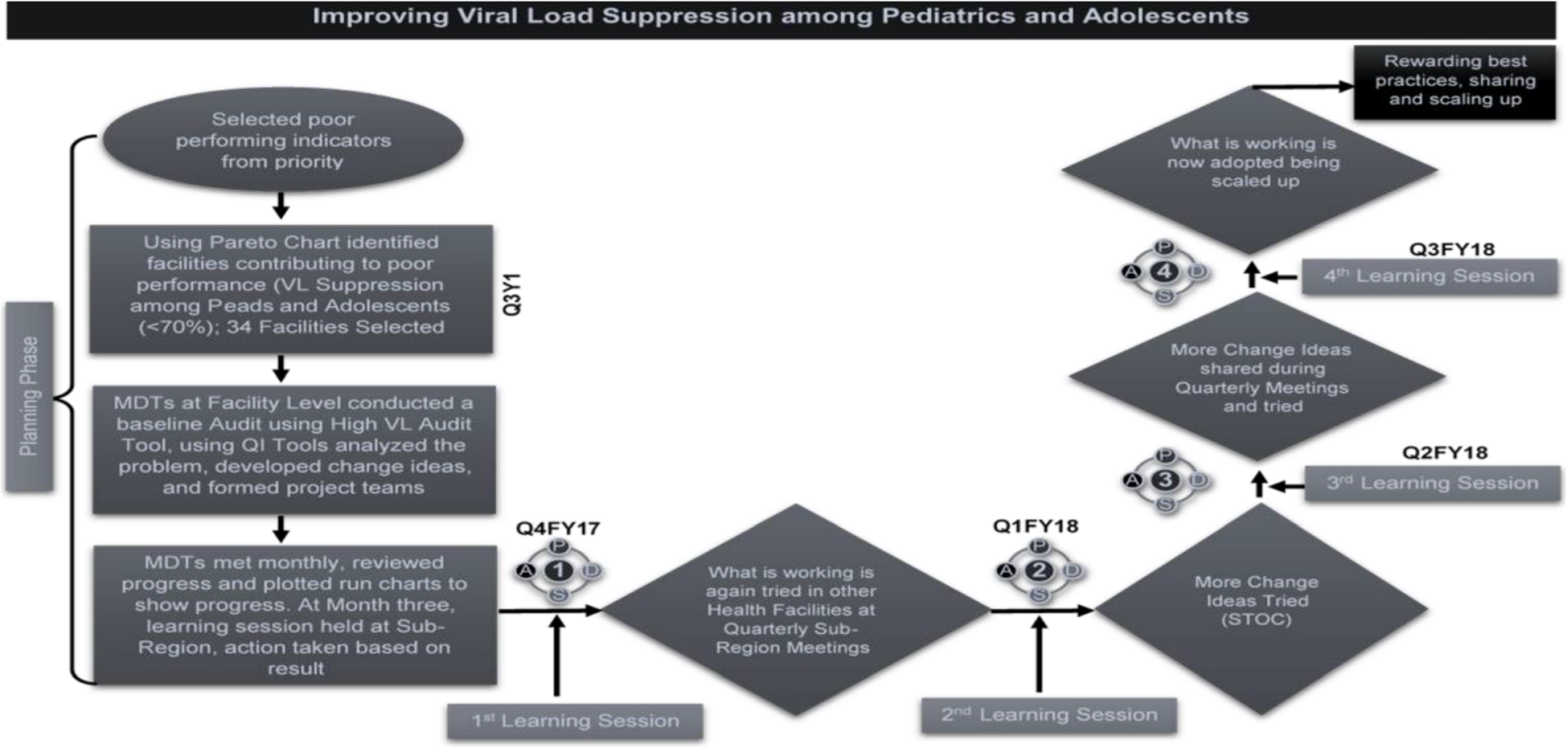
CQI processes for improving viral load suppression among adolescents

We adopted CQI approaches to improve performance in 65 selected health facilities in Western Kenya, which initiated changes to improve the viral load testing uptake. The process involved quarterly learning sessions and an award/harvesting session focused on recognizing all the CQI teams at the end of the project (Fig. 6). We also identified facility-based viral load champions, involved caregivers during barrier analysis sessions, identified surrogate caregivers, profiled adolescents and initiated differentiated service delivery, including weekend clinics and peer drug delivery to boarding schools. Stamping was introduced to confirm sample collection, and clients who resuppressed were celebrated.

##### Outcomes

**Fig. 7:**
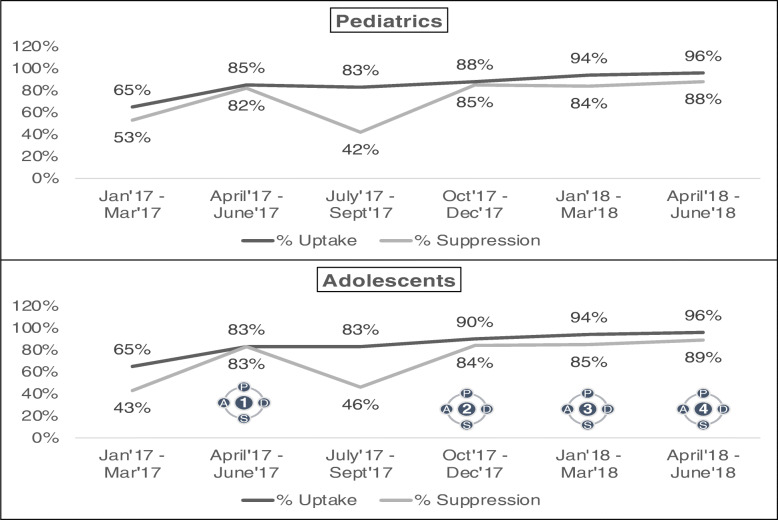
Uptake and percentage suppression among paediatrics and adolescents

The viral load testing uptake among paediatric and adolescent patients improved from a low of 65 % to a high of 96 %, while suppression improved from a low of 53 % to a high of 88 % (Fig. 7).

**Fig. 8:**
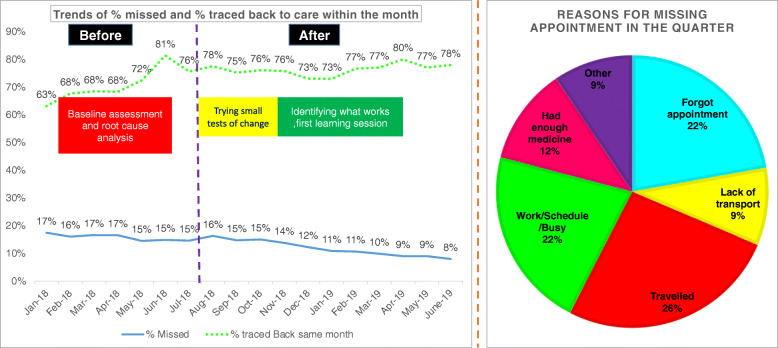
Trends of missed and tracked care appointments and reasons for missing appointments

Additionally, retention in care was identified as one of the key areas for improvement. Using the Pareto principle, 58 facilities in Western Kenya that contributed to program losses in the previous year were identified. These facilities started a CQI collaborative to improve the processes that aimed to reduce their loss to follow-up. The aim of the collaborative was to reduce the number of clients who missed their appointments and to increase the number of clients who could be traced back to care within 30 days of missing appointments. After implementing the CQI processes, the proportion of clients missing appointments decreased from 17 to 8 %, while that of clients who could be traced back to care increased from 63 % to a high of 78 % (Fig. 8).

## Discussion

We present country case studies showing that institutionalizing CQI approaches in HIV service delivery improved outcomes along the 95:95:95 cascade. Through our CQI approach, we were able to increase PNS outcomes and the capacity of health providers and community systems in HIV identification to encourage more partner-initiated counselling for HIV testing. This experience showed that despite overstretched health systems, economic constraints, limited or poor training and inadequate laboratory infrastructure [19], facilities can apply CQI approaches and learn to use data to review their performances and make changes to improve their quality of care. The facilities described in the first case study achieved the aim of identifying more HIV-positive individuals, connecting them with the healthcare system, and getting them enrolled in HIV services, which contributed to overall improvements in HIV treatment with a reduction in the risk of transmission. Individuals who receive care earlier can help with HIV testing, knowledge of status and, when positive, the immediate initiation of treatment, in addition to building a fruitful foundation for the provider–client relationship [20].

Our CQI approach also showed improvements in linkage to HIV care and treatment. In this case, we used community-based volunteers to immediately link HIV-positive patients and initiate them into treatment. We also implemented structured, contextual practices to ensure that immediate follow-up during the first few weeks was enhanced. Working together with lay health workers has helped reach more households and identify individuals at risk for HIV who were previously unknown to healthcare workers [21–23]. However, linking community-level interventions with improved health outcomes remains a challenge. In the third case study, we show facilities that successfully increased VS among paediatrics during the time that they used CQI approaches, which helped individuals remain in care and ensured that appropriate services were delivered during clinic visits.

Our experience offers a model that can be adopted, scaled up and built upon to improve HIV service delivery and quality of care, especially in LMICs. Using our approach, the key elements of success were (1) breaking down problems into smaller, more specific components; (2) addressing those problems with data-driven CQI approaches led by subnational and facility-based CQI teams; (3) multistakeholder, in-country leadership; (4) on-site CQI coaching; and (5) inter- and intracountry shared learning and support through CQI collaboratives.

### Limitations

However, these findings need to be interpreted in the context of several limitations. First, we do not share country-specific data beyond aggregate data, although the trends are deemed adequate for informing CQI through evaluation and learning as opposed to the robust granular data needed to inform research. Second, we do not identify individual facilities, partly because the respective MoHs are understandably cautious about data being used to judge specific facilities compared to others. Since the country-level data are owned by the MoH, it is important to blind the facilities in the dissemination of our data. However, since data review and sharing is a core requirement of the CQI process, we were able to overcome this limitation in real time, as well as to provide summative cross-country learning. Finally, as previously described, different countries had divergent approaches, which may have led to divergent applications of the CQI approach and variations in the strength of our results.

## Conclusions

We present select case study findings from high-HIV-epidemic sub-Saharan African countries to showcase strategies for meeting global HIV targets along the 95:95:95 cascade using CQI approaches. Our Collaborative CQI model improved HIV outcomes in high HIV burden LMIC settings of Rwanda, Botswana and Kenya. Similarly designed CQI initiatives could increase MOH ownership through joint design and participation as CQI coaches resulting in health systems strengthening and sustainable implementation. The described CQI model can be easily adapted or adopted for all settings for accelerating quality improvement, learning and client-centred outcomes.

## Data Availability

The datasets generated and/or analysed during the current study are not publicly available as they were used for health improvement purposes; however, they are available from the corresponding author upon reasonable request, with permission from the Ministry of Health in the respective countries.

## Abbreviations

ART: Anti-retroviral Treatment
CQI: Continuous Quality Improvement
ECHO: Extension for Community Healthcare Outcomes
HTS: HIV Testing Services
LMIC: Low and Middle Income Countries
MoH: Ministry of Health
PDSA: Plan Do Study Act
PNS: Partner Notification Services
